# Psychometric properties of the critical thinking disposition assessment test amongst medical students in China: a cross-sectional study

**DOI:** 10.1186/s12909-020-02437-2

**Published:** 2021-01-06

**Authors:** Liyuan Cui, Yaxin Zhu, Jinglou Qu, Liming Tie, Ziqi Wang, Bo Qu

**Affiliations:** 1 Institute for International Health Professions Education and Research, China Medical University, Shenyang, 110122 China; 2 China Medical University Library, China Medical University, Shenyang, 110122 China; 3 Department of Postgraduate Administration, The First Hospital of China Medical University, Shenyang, 110122 China; 4 The Third Clinical Department, China Medical University, Shenyang, 110122 China

**Keywords:** Critical thinking disposition, Medical students, China, Critical thinking disposition assessment (CTDA), Reliability, Validity

## Abstract

**Background:**

Critical thinking disposition helps medical students and professionals overcome the effects of personal values and beliefs when exercising clinical judgment. The lack of effective instruments to measure critical thinking disposition in medical students has become an obstacle for training and evaluating students in undergraduate programs in China. The aim of this study was to evaluate the psychometric properties of the CTDA test.

**Methods:**

A total of 278 students participated in this study and responded to the CTDA test. Cronbach’s α coefficient, internal consistency, test-retest reliability, floor effects and ceiling effects were measured to assess the reliability of the questionnaire. Construct validity of the pre-specified three-domain structure of the CTDA was evaluated by explanatory factor analysis (EFA) and confirmatory factor analysis (CFA). The convergent validity and discriminant validity were also analyzed.

**Results:**

Cronbach’s alpha coefficient for the entire questionnaire was calculated to be 0.92, all of the domains showed acceptable internal consistency (0.81–0.86), and the test-retest reliability indicated acceptable intra-class correlation coefficients (ICCs) (0.93, *p* < 0.01). The EFA and the CFA demonstrated that the three-domain model fitted the data adequately. The test showed satisfactory convergent and discriminant validity.

**Conclusions:**

The CTDA is a reliable and valid questionnaire to evaluate the disposition of medical students towards critical thinking in China and can reasonably be applied in critical thinking programs and medical education research.

## Background

For decades, the importance of developing critical thinking skills has been emphasized in medical education [1]. As listed by the World Federation for Medical Education, critical thinking should be part of the training standards for medical students and practitioners [2]. Critical thinking is essential for medical students and professionals to be able to evaluate, diagnose and treat patients effectively [3]. One major criticism of medical education is the gap that exists between what students learn in the classroom setting and what they experience in clinical practice [4]. Only a few students will analyze and employ critical thinking when they acquire knowledge during their education [5]. Therefore, critical thinking has become increasingly necessary for medical students and professionals [6].

Critical thinking is an indispensable component of ethical reasoning and clinical judgment, and possessing reasonable critical thinking abilities reduces the risk of clinical errors [7]. Adverse events that occur by human error and preventable medical errors were frequently caused by a failure of cognitive function (e.g., failure to synthesize and/or take action based on information), which was second only to ‘failure in technical operation of an indicated procedure’ [8, 9]. Similar problems have been reported in several countries such as the United Kingdom, Canada and Denmark [10]. Therefore, medical professionals need to exercise critical thinking, transcend simple issues, and make sound judgments in order to handle adverse medical situations [11]. Providing evidence and logical arguments to medical students and professionals is beneficial in order to support clinical decision-making and assertions [12]. Lipman and Deatrick are of the same opinion; i.e., critical thinking is a prerequisite for sound clinical decision-making [13]. Therefore, medical students should be exposed to clinical learning experiences that promote the acquisition of critical thinking abilities that are needed to provide quality care for patients in modern complex healthcare environments [14].

Currently, critical thinking is defined as a kind of reasonable reflective thinking following the synthesis of cognitive abilities and disposition [15]. The former includes interpretation, deduction, induction, evaluation and inference, whereas the latter includes having an open mind and being intellectually honest [16]. The critical thinking disposition (CTD) was described as seven attributes including, truth-seeking, open-mindness, analyticity, systematicity, critical thinking self-confidence, inquisitiveness and maturity [17]. A disposition to critical thinking is essential for professional clinical judgement [18]. An assessment of the CTD in professional judgment circumstances and educational contexts can establish benchmarks to advance critical thinking through training programs [4].

To investigate and assess the CTD in medical students, a reliable and valid tool is indispensable. Several CTD measurement tools are available, such as the California Critical Thinking Dispositions Inventory (CCTDI), Yoon’s Critical Thinking Disposition (YCTD) and the Critical Thinking Disposition Assessment (CTDA). The CCTDI was developed to evaluate the CTD in normal adults. It had good reliability and validity in western cultures, however, it had low reliability and validity for Chinese nursing students in previous studies [19, 20]. Yoon created the YCTD, which was based on the CCTDI, for nursing students in South Korea [21]. According to the literature review and other measures of critical thinking disposition, Yuan developed the CTDA in English version. They used it to measure the CTD for medical students and professionals. In his study, the Cronbach’s alpha for the entire assessment was 0.94 [22]. The CTD for the CTDA were defined as “systematicity and analyticity”, “inquisitiveness and conversance” and “maturity and skepticism”. The “systematicity and analyticity” portion is the cognitive component of the CTD and measured the tendency towards organizing and applying evidence to address problems. Being systematical and analytical allow medical students to connect clinical observations with their knowledge to anticipate events that are likely to threaten the patient’s safety [23]. The “inquisitiveness and conversance” is the motivation component of the CTD. It measures the desire of medical students for learning whenever the application of the knowledge is inconclusive and is essential for medical students to expand their knowledge in clinical practice [24]. The “maturity and skepticism” is the personality component of the CTD which measured the disposition to be judicious in decision making and how often it leads to reflective skepticism. This disposition has particular implications for ethical decision making, particularly in time-pressured clinical situations [25]. All the domains have a tight connection to one another. In adapting to the Chinese version, we followed the translation and cross-cultural adaptation of the guidelines set forth by the WHO [26]. The steps listed by the WHO are as follows: forward translation, expert panel review, back translation, pretest and cognitive interviews, and formulation of the final version. As such, the CTDA may be especially valuable for institutes or universities in Asian countries or with an Eastern culture for assessing critical thinking disposition in medical students. Given the lack of effective instruments to assess the CTD in undergraduate medical programs in mainland China, the objective of this investigation was to evaluate the psychometric properties of the CTDA.

## Methods

### Sample sizes

According to Kline’s recommendation, it is necessary to note that the sample size should base on the principle of a 1:10 item to participant ratio [27]. The total number of items in the CDTA is nineteen and so the sample size should be at least 190 students. Therefore, using this guideline, with a sample size of 300 students, this research exceeds the recommended minimum.

### Participants and procedures

Students of clinical medicine in China must undergo 5 years of medical training. Years 1 and 2 are dedicated to the basic sciences, years 3 and 4 to clinical medicine, and year 5 is the clinical internship. This study involved stratified-cluster random sampling. Firstly, the participants were recruited from different academic years. Two classes were selected randomly from each year. There were approximately 30 individuals in one class, with 300 medical students enrolled in this study in total. The sample of the study for test-retest reliability to assess the ICCs is 14 [28]. Forty-nine respondents were randomly selected to finish the online survey 2 weeks later and 43 participants completed it.

Three hundred medical students completed the online survey between March and June 2019. Respondents provided written consent to participate in the study. A self-administered questionnaire was applied in the survey. The anonymity of participants was guaranteed and all of students voluntarily took part in the study. It took approximately 15 to 20 min to submit the questionnaire.

### Instrument

The questionnaire consisted of two components: part A which included sociodemographic characteristics (e.g., age, gender, and academic year) and part B which contained the CTDA. The CTDA assessed the CTD of medical students and professionals and was comprised of 19 items in three domains as follows: “systematicity and analyticity”, “inquisitiveness and conversance”, and “maturity and skepticism”. Items were rated on a seven-point Likert scale ranging from 1 to 7 (1 for very *strongly disagree* and 7 for very *strongly agree*) [22]. Each domain was computed to the sum of its item score and the total CTDA was calculated by the sum of its domain scores. Higher scores signified higher CTD.

### Statistical analysis

#### Reliability

We computed Cronbach’s α as a measure of internal consistency along with the means, standard deviation, skewness, kurtosis, ceiling and floor effects of the questionnaire and its domains. Absolute values of skewness and kurtosis higher than 3 and 10 respectively showed significant deviance from a normal subject’s distribution [29]. Student’s F-test was performed to determine the association between the academic year and domains of the CTDA. The ceiling and floor effects were considered abnormal when the highest/lowest scores were higher than 20% [30, 31]. Following Kline’s recommendations, a Cronbach’s alpha above 0.70 was considered satisfactory [27]. The test-retest reliability was good if the ICC was higher than 0.70.

#### Validity

With the purpose of assessing construct validity, the original three-factor structure of the CTDA was applied for explanatory factor analysis (EFA) and confirmatory factor analysis (CFA). Factor analysis using principal component analysis with direct oblimin rotation was used and a factor load > 0.4 was considered as acceptable [32]. Domains of the instrument was assessed based on selected criteria through the following indexes: a) CMIN/DF < 3; b) RMSEA< 0.08; c) AGFI> 0.80; d) the *p* value should be significant [33, 34]. Pearson’s correlation coefficient between each domain of the CTDA was used to test the inter-correlation of the scale.

The convergent and discriminant validity of the questionnaire was measured by computing item-domain Pearson’s correlations. If the former was more than 0.4, it indicated that the items and their domains were acceptable [35]. The latter was considered satisfactory if items showed correlations with other domains that were lower than those with their own domains. The CFA was conducted with AMOS 21 and other statistics were calculated with SPSS 23. Ten students checked the face validity. Each item received positive feedback from students indicating that the CTDA had good face validity.

## Results

### Basic characteristics of the study sample

Of the total number of 300 students participating in the research, 278 (92.67%) completed the study. The mean age of the 278 individuals was 20.88 ± 1.76 years (SD); within the study sample, 113 of the participants (40.64%) were male. Additionally, 54 of the individuals (19.42%) were first year students and 55 of the students (19.78%) were fifth year students.

### Score distributions

Across domains, “systematicity and analyticity” obtained the highest score (43.93 ± 5.71), whereas “maturity and skepticism” scored the lowest (28.41 ± 3.96). The skewness and kurtosis coefficients of the entire questionnaire were acceptable, with the former ranging from − 0.98 to − 0.32 and the latter ranging from − 0.13 to 2.05. There were no floor effects in the three domains. However, items 12, 18, and 19 showed significant ceiling effects ranging from (20.14–23.74%).

### Reliability

The overall Cronbach’s α coefficient of the CTDA was good (0.92) and showed good internal consistency. The three domains were considered to have shown acceptable internal consistency (0.81–0.86). The overall split-half reliability coefficient of the CTDA was acceptable (0.89). The retest response rate was 83.67% (41/49), and the test-retest reliability (0.93) revealed statistically significant ICCs for the three domains. In addition, the Pearson’s correlation coefficients of all domains were acceptable. The results are reported in Table 1.

**Table 1:** Reliability of the CTDA

	Cronbach’s α coefficient(***n*** = 278)	ICCs (95%CI) (***n*** = 43)
Domains		
Systematicity and analyticity	0.86	0.72(0.36–0.79) ^**^
Inquisitiveness and conversance	0.81	0.71(0.34–0.81) ^**^
Maturity and skepticism	0.85	0.79(0.39–0.85) ^**^
CTDA	0.92	0.79(0.34–0.81) ^**^

^**^p < 0.01

*ICCs* intraclass correlation coefficients, *CTDA* the critical thinking disposition assessment

### Validity

#### Construct validity

##### EFA

The Kaisex-Meyer-Olkin test result was 0.92 and Bartlett’s test of sphericity yielded a *p* < 0.05, signifying that the gathered results indicated that factor analysis could be performed. The EFA revealed factors with eigenvalues greater than 1, accounting for 57.13% of the variance. A three-factor solution based on the results was reported in the rotated component matrix (Table 2).

**Table 2:** Rotated component matrix

Item	Factor		
	Systematicity and analyticity	Inquisitiveness and conversance	Maturity and skepticism
1	**0.64**	0.17	0.11
2	**0.76**	0.06	0.30
3	**0.66**	0.32	0.17
4	**0.50**	0.34	0.33
5	**0.59**	0.33	0.14
6	**0.63**	0.06	0.25
7	**0.62**	0.08	0.40
8	**0.65**	0.23	0.30
9	0.04	**0.77**	0.18
10	0.31	**0.51**	0.49
11	0.28	**0.71**	0.35
12	0.56	**0.44**	0.22
13	0.37	**0.60**	0.03
14	0.42	**0.42**	0.46
15	0.23	0.23	**0.58**
16	0.27	0.11	**0.82**
17	0.24	0.11	**0.79**
18	0.16	0.13	**0.74**
19	0.24	0.20	**0.75**

Items are bolded per column to indicate the relevant factor in which they belong

##### CFA

**Fig. 1:**
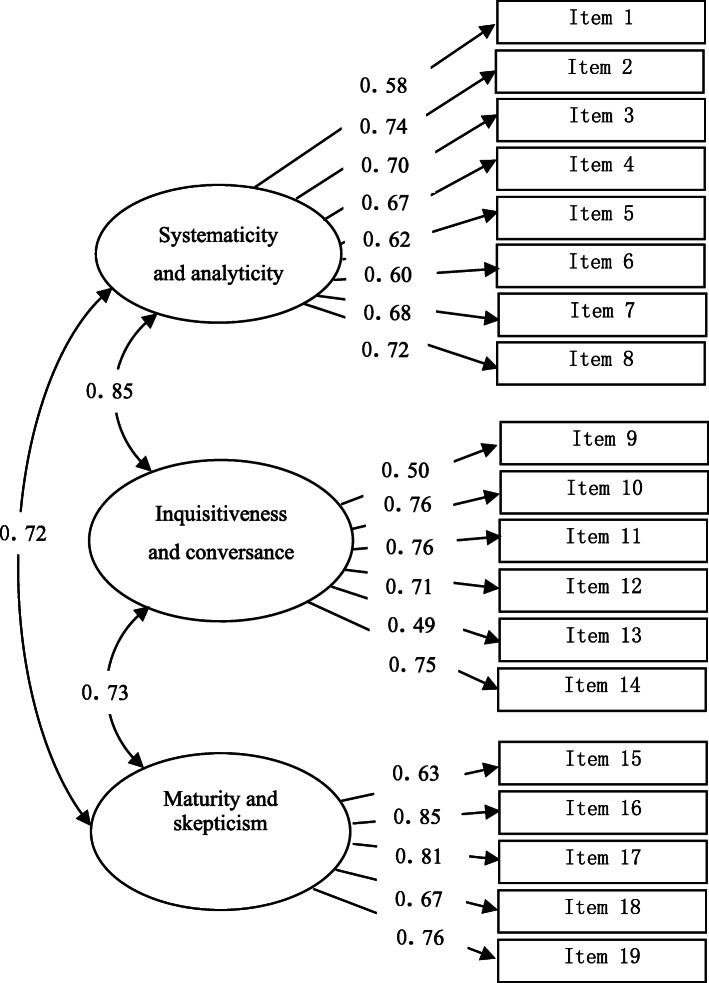
The model of the CTDA based on CFA. CTDA, the critical thinking disposition assessment

We performed a CFA of the three-factor structure with 19 items to demonstrate that the structure showed an acceptable fit with the data (χ2 = 410.75, df = 149, CMIN/DF = 2.76, CFI = 0.90, AGFI = 0.83, *p* < 0.05, RMSEA = 0.08 [90% CI: 0.07 to 0.09]). Factor loadings were higher than 0.40 and ranged from (*r =* 0.50–0.85), as illustrated in Fig. 1.

#### Correlation analysis between CTDA domains

The CTDA showed significant correlation between any of the two assessment domains (*r =* 0.61–0.72), with *p* values less than 0.01. The correlations between assessment domains based on Pearson’s correlation are shown in Table 3.

**Table 3:** Correlations between the CTDA domains(*n* = 278)

Domain	Systematicity and analyticity	Inquisitiveness and conversance	Maturity and skepticism
Systematicity and analyticity	–	0.72^**^	0.61^**^
Inquisitiveness and conversance	0.72^**^	–	0.61^**^
Maturity and skepticism	0.61^**^	0.61^**^	–

^**^p < 0.01

*CTDA* the critical thinking disposition assessment

#### Convergent validity and discriminant validity

Based on item-domain correlations, the scores of each item correlated with their own domain to an acceptable degree (*r =* 0.65–0.86, *p* < 0.01), and the convergent validity of the CTDA was acceptable. In addition, whole items showed a higher correlation with their own domains than with other domains and the discriminant validity was satisfactory, as shown in Table 4.

**Table 4:** Convergent and discriminant validity of the CTDA (*n* = 278)

Domain	Correlation coefficient range		Convergent validity		Discriminant validity	
	Convergent validity	Discriminant validity	Success/total	Percentage (%)	Success/total	Percentage (%)
1	0.66–0.79^**^	0.36–0.58^**^	8/8	100	8/8	100
2	0.65–0.81^**^	0.27–0.63^**^	6/6	100	6/6	100
3	0.72–0.86^**^	0.44–0.55^**^	5/5	100	5/5	100

^**^p < 0.01

*CTDA* critical thinking disposition assessment

#### Dose-response analysis

The relationship between the academic year and domains of the CTDA is reported in Table 5. It indicated that there were significant differences among the 5 years on the CTDA score and domains. The year 2 students obtained the highest of the CTDA (107.88 ± 11.34) and the year 1 students scored 107.20 ± 12.14. Surprisingly, the year 5 students reported the lowest level at (98.91 ± 12.52). Among the 5 years, the year 2 students had the highest score (45.32 ± 5.01) in “systematicity and analyticity” and the year 1 students obtained the highest score (33.76 ± 4.59) in “inquisitiveness and conversance”. Moreover, the highest scores overall were the year 3 students’ in “maturity and skepticism” at 29.16 ± 3.47. On the other side, the year 5 students had the lowest scores in all of the domains.

**Table 5:** Relationship between the academic year and domains of the CTDA (*n* = 278)

Variable	N(%)	Systematicity and analyticity	F	Inquisitiveness and conversance	F	Maturity and skepticism	F	CTDA	F
Academic year^a^									
1	54 (19.4)	44.89 ± 5.33	3.79*	33.76 ± 4.59	3.98*	28.56 ± 3.85	3.79*	107.20 ± 12.14	4.70*
2	59 (21.2)	45.32 ± 5.01		33.49 ± 4.84		29.07 ± 3.16		107.88 ± 11.34	
3	55 (19.8)	43.95 ± 5.44		32.42 ± 4.31		29.16 ± 3.47		105.53 ± 11.23	
4	55 (19.8)	43.87 ± 6.48		32.95 ± 4.42		28.60 ± 4.52		105.42 ± 13.83	
5	55 (19.8)	41.55 ± 5.67		30.71 ± 4.30		26.65 ± 4.31		98.91 ± 12.52	

* *p* < 0.05

^a^One-way ANOVA

*CTDA* the critical thinking disposition assessment

## Discussion

The psychometric properties of the questionnaire were satisfactory. Results demonstrated that the CTDA is good, reliable, and valid for Chinese medical students. In addition, all items and domains showed acceptable kurtosis and skewness coefficients. Our results were similar to those of previous studies conducted in Ireland and Iran using other critical thinking disposition instruments [36, 37]. However, three items showed a significant ceiling effect, above the accepted threshold of 20%. This result was comparable to that reported in two critical thinking studies which showed evidence of a ceiling effect in overall scores in the United States and China [38, 39]. The ceiling effect might be attributable to the population distribution at schools or universities [39].

It is clear that the domains of the CTDA showed rationally acceptable reliability when evaluating the CTD of medical students. The satisfactory Cronbach’s α coefficient values of the domains demonstrate the high internal consistency of the entire questionnaire. Our results are in line with other studies conducted in Asian countries, as seen by the Cronbach’s α reliability of the CCTDI of 0.87 in Turkey by Iskifoglu [40] and 0.80 in Iran by Gupta [41]. Our study showed the Cronbach’s alpha of the CTDA was 0.92, which was similar to the value reported in the original study [22]. Therefore, the Cronbach’s α indicates that the whole internal reliability of the CTDA test is satisfactory.

Our findings indicated that the EFA of the CTDA conducted with medical students and professionals yielded a three-domain model. The EFA model of our study was the same to the previous study [22]. Our CFA results indicated that the three-factor structure (“systematicity and analyticity”, “inquisitiveness and conversance” and “maturity and skepticism”) of the CTDA (AGFI = 0.83, RMSEA = 0.08) showed an acceptable fit with the data. It is likely that the differences in the domains depend on the different theoretical models [42]. The domains of the CTDA test were similar to those reported in other studies conducted in Asian countries like Turkey, Japan and Korea based on the theoretical model of Facione. Yoshinori reported that the CFA of the CTD Scale displayed four subfactors, similar to our study [43]. Shin noted that the CFA of the YCTD revealed a seven-domain model, and three of the domains (systematicity, intellectual eagerness/curiosity, and healthy skepticism) were similar to those of our study [44]. However, Zuriguelperez reported that the CFA of the Critical Thinking Questionnaire completed by Spanish students yielded a four-factor model (personal, intellectual and cognitive, interpersonal/self-management and technical), based on the Alfaro-LeFevre theoretical model [45].

Similar results were found by Yuan and Wang’s studies in the critical thinking disposition inventory for Chinese medical students [6, 22]. Our research offers a plausible explanation for the high correlations between the domains. “Inquisitiveness and conversance” could be taken to mean that the students have the desire for learning and are intellectually curious while “systematicity and analyticity” could mean that students use reason and evidence to address problems with systemic thinking. Both of them have a tight connection with one another.

Our research demonstrated that the convergent validity and discriminant validity of the CTDA were satisfactory and all items displayed a higher correlation with their own domain than with other domains. Therefore, no items need to be modified or reassigned to another domain. Other studies conducted in China have reported similar results in terms of convergent and discriminant validity of the CTD instrument [46, 47].

We reported that the CTD scores of the year 5 medical students were lower than those of the year 1 students. The explanation could be that employment pressure and the stress of internship for the fifth-year students may have made their CTD worse. In addition, Ip suggested that the CTD scores of the younger Chinese nursing students were higher than the older students, especially in the domain between inquisitiveness and confidence [19]. Similar result was found by Kim in Korean nursing students. They found that the domain scores between intellectual integrity and truth seeking in year 1 were higher than year 4 [48]. However, Hunter found that the CTD were the highest during the year 4 for nursing students [49].

The CTDA shows promise as an instrument for future studies on the CTD by medical students in China. However, certain limitations of our research should be acknowledged. First, the medical students were recruited from a single medical institution in China, so the sample representativeness was limited. Second, due to time constraints, the findings of our study were limited by the size of the study population. Future studies could increase the representativeness of the study population by expanding sample diversity and size. Third, the concurrent validity of CTDA was not tested due to the lack of a widely used CTD scale. Fourth, the CTDA could only measure the dispositions or traits of critical thinking which cannot assess for critical thinking skills.

## Conclusions

Our findings demonstrate promising applicability of the CTDA, since the questionnaire is of good reliability and validity to measuring the CTD amongst Chinese medical students. The results may be valuable to other institutions involved in assessing critical thinking disposition in students.

## Data Availability

The datasets used and/or analysed during the current study available from the corresponding author on reasonable request.

## Abbreviations

CMIN/DF: Chi square divided by degrees of freedom
ICCs: Intra-class correlation coefficients
SD: Standard deviation
CFA: Confirmatory factor analysis
CTDA: The critical Thinking Disposition Assessment
RMSEA: Root mean square error of approximation
YCTD: Yoon’s Critical Thinking Disposition
AGFI: Adjusted goodness-of-fit index
No.: Number
CCTDI: California Critical Thinking Dispositions Inventory
CFI: Comparative fit index
EFA: Explanatory factor analysis
CTD: Critical thinking disposition

## References

[1] Monteiro S, Sherbino J, Sibbald M, Norman G (2020). Critical thinking, biases and dual processing: The enduring myth of generalisable skills. Med Educ.

[2] Schwarz MRWA (2002). Global minimum essential requirements: a road towards competence-oriented medical education. Med Teach.

[3] Ali-Abadi T, Babamohamadi H, Nobahar M (2020). Critical thinking skills in intensive care and medical-surgical nurses and their explaining factors. Nurse Educ Pract..

[4] Sharples J, Oxman AD, Mahtani KR, Chalmers I, Oliver S, Collins K, Austvolldahlgren A, Hoffmann T (2017). Critical thinking in healthcare and education. BMJ..

[5] Paul R, Elder L (2013). Critical Thinking: Intellectual standards essential to reasoning well within every domain of human thought,part two. JDE.

[6] Wang X, Sun X, Huang T, He R, Hao W, LJBME Z (2019). Development and validation of the critical thinking disposition inventory for Chinese medical college students (CTDI-M). BMC Med Educ.

[7] Zayapragassarazan Z, Menon V, Kar S, Batmanabane G (2016). Understanding critical thinking to create better doctors. Adv Med Educ Pract.

[8] Reason JT (2000). Human error : models and management. BMJ.

[9] Croskerry P (2003). The importance of cognitive errors in diagnosis and strategies to minimize them. Acad Med.

[10] Wilson RM, Der Weyden MBV (2005). The safety of Australian healthcare: 10 years after QAHCS. Med J Aust.

[11] Beyer B (1985). Critical Thinking: what is it?. Soc Educ.

[12] Macpherson K, Owen C (2010). Assessment of critical thinking ability in medical students. Assess Eval High Educ.

[13] Lipman TH, Deatrick JA (1997). Preparing advanced practice nurses for clinical decision making in specialty practice. Nurse Educ.

[14] Senita J. Defining critical thinking experiences of senior nursing students.**[**Doctor's thesis]: Kent State University College; 2017.

[15] Facione NC, Facione PA (1996). Externalizing the critical thinking in knowledge development and clinical judgment. Nurs Outlook.

[16] Papp KK, Huang G, Clabo LML, Delva D, Fischer MA, Konopasek L, Schwartzstein RM, Gusic ME (2014). Milestones of critical thinking: a developmental model for medicine and nursing. Acad Med.

[17] Facione PA. Critical thinking: a statement of expert consensus for purposes of educational assessment and instruction(the Delphi report): The California Academic Press; 1990.

[18] Mann N (2018). Application of clinical judgment models to the development of academic advisors. NACADA J.

[19] Ip WY, Lee DTF, Lee IFK, Chau JPC, Wootton YSY, Chang AM (2000). Disposition towards critical thinking: a study of Chinese undergraduate nursing students. J Adv Nurs.

[20] Yeh ML (2002). Assessing the reliability and validity of the Chinese version of the California critical thinking disposition inventory. Int J Nurs Stud.

[21] Shin H, Park CG, Kim H (2015). Validation of Yoon's Critical Thinking Disposition Instrument. Asian Nurs Res (Korean Soc Nurs Sci).

[22] Yuan S, Liao H, Wang Y, Chou M (2014). Development of a scale to measure the critical thinking disposition of medical care professionals. Soc Behav Personal.

[23] Facione NC, Facione PA, Sanchez CA (1994). Critical thinking disposition as a measure of competent clinical judgment: the development of the California Critical Thinking Disposition Inventory. J Nurs Educ.

[24] Palmer S. Critical thinking dispositions of part-time faculty members teaching at the college level.[Doctor's thesis]: The College of Education of Ohio University; 2007.

[25] Costanzo FJ. The impact of a standardized participant simulation learning experience on the critical thinking disposition of undergraduate health administration students.[Doctor's thesis]: Rowan University; 2015.

[26] World Health Organization[WHO]. Process of translation and adaptation of instruments. Available from: http://www.who.int/substance_abuse/research_tools/translation/en/.Accessed 11 Jan, 2019.

[27] Kline P (2000). The handbook of psychological testing.

[28] Shoukri M, Asyali MH, Donner A (2004). Sample size requirements for the design of reliability study: review and new results. Stat Methods Med Res.

[29] Ferreira-Valente A, Costa P, Elorduy M, Virumbrales M, Costa MJ, Palés J (2016). Psychometric properties of the Spanish version of the Jefferson Scale of Empathy: making sense of the total score through a second order confirmatory factor analysis. BMC Med Educ.

[30] Cisneros RM (2009). Assessment of critical thinking in pharmacy students. Am J Pharm Educ.

[31] Zhu Y, Liu J, Qu B (2017). Psychometric properties of the Chinese version of the WHOQOL-HIV BREF to assess quality of life among people living with HIV/AIDS: a cross-sectional study. BMJ Open.

[32] Rovine MJ (2000). Molenaar PC.A structural modeling approach to a multilevel random coefficients model. Multivar Behav Res.

[33] Kline R (2011). Principles and practice of structural equation modeling.

[34] Wu ML (2017). Operation and application of structural equation model Amos.

[35] Reychler G, Caty G, Vincent A, Billo S, Yombi JC (2013). Validation of the French version of the World Health Organization quality of life HIV instrument. PLoS One.

[36] Noone C, Bunting B, Hogan M (2016). Does Mindfulness enhance critical thinking? evidence for the mediating effects of executive functioning in the relationship between mindfulness and critical thinking. Front Psychol.

[37] Yaghoubi A, Habibinejad P (2015). Teacher burnout and critical thinking among EFL teachers. JEMS.

[38] Smith LS, Gillette C, Taylor SR, Manolakis M, Dinkins MM, Ramey C (2019). A semester-long critical thinking course in the first semester of pharmacy school: Impact on critical thinking skills. Curr Pharm Teach Learn.

[39] Yi CQ. Study on the American "collegiate learning assessment(CLA+)" [Master's thesis]: Yunnan University; 2018.

[40] Iskifoglu G (2013). Cross-cultural equivalency of the California critical thinking disposition inventory. Educ Sci Theory Pract.

[41] Gupta K, Iranfar S, Iranfar K, Mehraban B, Montazeri N (2012). Validly and reliability of California critical thinking disposition inventory (CCTDI) in Kermanshah University of Medical Sciences. Educ Res Med Sci.

[42] Devellis R (2016). Scale development: theory and applocations.

[43] Sugiura Y (2013). The dual effects of critical thinking disposition on worry. PLoS One.

[44] Shin K, Jung DY, Shin S, Kim MS (2006). Critical thinking dispositions and skills of senior nursing students in associate, baccalaureate, and RN-to-BSN programs. J Nurs Educ.

[45] Zuriguelperez E, Falcopegueroles A, Roldanmerino J, Agustinorodriguez S, Gomezmartin MDC, Lluchcanut MT (2017). Development and psychometric properties of the nursing critical thinking in clinical practice questionnaire. Worldviews Evid-Based Nurs.

[46] Liu H. The structure, measurement and development characteristics of teenagers’ critical thinking disposition. [Master's thesis]: Wenzhou University; 2018.

[47] Wang L.The revise and testing of the self-designed questionnaire of college students’ critical thinking. [Master's thesis]. Shantou University,2011.

[48] Kim DH, Moon S, Kim EJ, Kim YJ, Lee S (2014). Nursing students' critical thinking disposition according to academic level and satisfaction with nursing. Nurse Educ Today.

[49] Hunter S, Pitt V, Croce N, Roche J (2014). Critical thinking skills of undergraduate nursing students: description and demographic predictors. Nurse Educ Today.

